# Beads‐on‐a‐Tip testing for ultrasensitive antigen detection across a large dynamic range

**DOI:** 10.1002/smo2.70036

**Published:** 2026-01-05

**Authors:** Ziwei Wu, Yangjian Cai, Yitong Zhao, Mahnaz Maddahfar, Mohammad Sadraeian, Dayong Jin, Jiajia Zhou

**Affiliations:** ^1^ Institute for Biomedical Materials and Devices (IBMD), Faculty of Science University of Technology Sydney Sydney New South Wales Australia; ^2^ ARC Centre of Excellence in Quantum Biotechnology (QUBIC), Institute for Biomedical Materials and Devices (IBMD), Faculty of Science University of Technology Sydney Sydney New South Wales Australia; ^3^ Zhejiang Provincial Engineering Research Center for Organelles Diagnostics and Therapy Eastern Institute of Technology Ningbo Zhejiang China

**Keywords:** Beads‐on‐a‐Tip, COVID‐19, rapid testing, ultrasenstive assay, upconversion nanoparticles

## Abstract

Lateral flow immunoassays (LFIAs) are low‐cost, rapid, and easy to use for point‐of‐care testing (POCT), but the majority of the available LFIA tests are indicative, rather than quantitative, and their sensitivity in antigen tests are usually limited at the nanogram range, which is primarily due to the passive capillary fluidics through nitrocellulose membranes, often associated with non‐specific bindings and high background noise. To overcome this challenge, we report a Beads‐on‐a‐Tip design by replacing nitrocellulose membranes with a pipette tip loaded with magnetic beads. The beads are pre‐conjugated with capture antibodies that support a typical sandwich immunoassay. This design enriches the low‐abundant antigen proteins and allows an active washing process to significantly reduce non‐specific bindings. To further improve the detection sensitivity, we employed upconversion nanoparticles (UCNPs) as luminescent reporters and SARS‐CoV‐2 spike (S) antigen as a model analyte to benchmark the performance of this design against our previously reported methods. We found that the key to enhance the immunocomplex formation and signal‐to‐noise ratio lay in optimizing incubation time and the UCNP‐to‐bead ratio. We therefore successfully demonstrated that the new method can achieve a very large dynamic range from 500 fg/mL to 10 μg/mL, across over 7 digits, and a limit of detection of 706 fg/mL, nearly another order of magnitude lower than the best reported LFIA using UCNPs in COVID‐19 spike antigen detection. Our system offers a promising solution for ultra‐sensitive and quantitative POCT diagnostics.

## Graphical abstract

Non‐specific adsorption on nitrocellulose membranes limits the sensitivity of conventional lateral flow immunoassays. Here, we present a Beads‐on‐a‐Tip immunoassay that replaces the membrane with magnetic beads for antigen capture and enrichment, while employing upconversion nanoparticles as background‐free luminescent reporters. Magnetic trapping within a pipette tip enables efficient washing, signal confinement, and reduced non‐specific binding. Combined with near‐infrared excitation, this platform achieves rapid and ultrasensitive antigen detection with a sub‐picogram‐level limit of detection, offering a robust alternative for point‐of‐care testing.

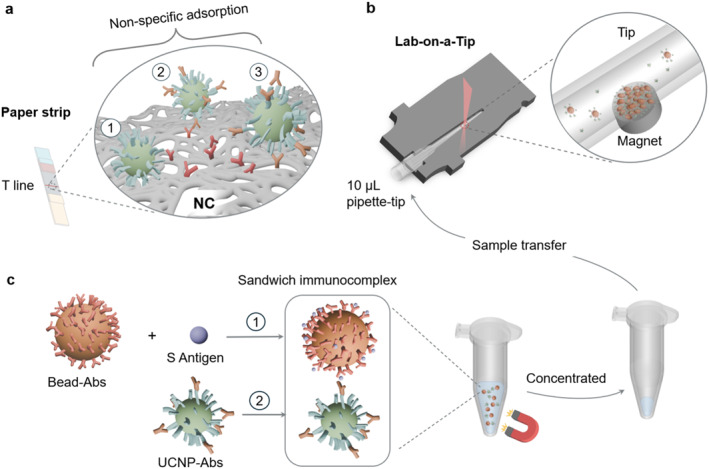

## 1 INTRODUCTION

**SCHEME 1:**
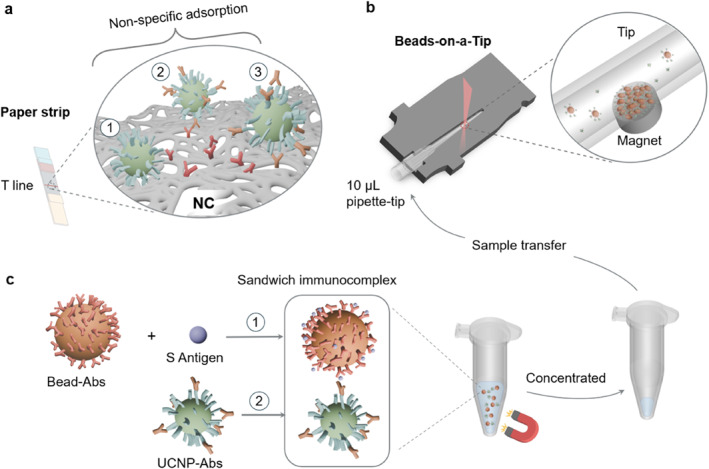
Schematic illustration of paper strip based lateral flow immunoassay versus Beads‐on‐a‐Tip immunoassay. (a) The three possible senarios for reporter nanoparticles (with and without antibodies) to form non‐specific bindings and therfore false positive signals at the testing line on the nitrocellulose membrane of lateral flow strip; (b) Beads‐on‐a‐Tip design, including a pipette tip embeded into a adapter with external deminsions identical to the strip cartridge we previously reported,^[6]^ a magnet to trap MBs and to enrich immunocomplexes, and a focused laser beam identical to what we previously used in the upconversion strip reader^[7]^; (c) Formation of a sandwich immunocomplex, followed by magnetic washing to remove unbound UCNPs and enrich analytes prior to being transferred into the pipette tip. MBs, magnetic beads; UCNPs, upconversion nanoparticles.

The COVID‐19 pandemic underscores the urgent need in rapid, accurate, and non‐invasive point‐of‐care testing (POCT). While polymerase chain reaction is highly accurate and widely regarded as the diagnostic gold standard,[^1^, ^2^] its time‐intensive protocol, dependence on sophisticated laboratory equipment, and need for skilled technicians restrict its practicality in POCT in clinical settings or at home. The World Health Organization recommended rapid antigen test (RAT) for emergency use, owing to their rapid turnaround time, affordability, and user‐friendly,^[3]^ but their limited sensitivity remains a concern, which is particularly true during the early stages of infection. RAT is based on lateral flow immunoassays (LFIAs) that typically rely on the porous membrane, that is, nitrocellulose membrane, to generate capillary flow, which drives the transport of reporter nanoparticles and facilitates their interaction with analytes at conjugation pad and with capture antibodies at the test line.[^4^, ^5^] LFIAs often suffer from the various forms of non‐specific adsorption of reporter nanoparticles at the test line, including nanoparticles with and without active antibodies, as illustrated in Scheme 1a, as non‐specific binding contributes to background noise, particularly when the analytes of target antigens are low‐abundant, for example, in the pico‐gram range.

Gold nanoparticles (AuNPs) are the most widely used nanoparticles as the signal reporter.[^8^, ^9^] When functionalized with specific antibodies, they selectively bind with the analyte of interest with color change. Luminescent nanoparticles, including europium nanoparticles with a size of 200–400 nm, have been used to improve the signal‐to‐noise ratio.[^10^, ^11^] Towards quantitative LFIA, we employed a range of highly doped upconversion nanoparticles (UCNPs), and with the purposely engineered testing devices,^[6]^ achieved the limit of detection (LOD) in the range of 10–100 pg in detecting antigen analytes.[^6^, ^7^, ^12^] Compared with traditional fluorescent dyes and conventional nanoparticles, UCNPs possess several unique optical advantages. Their luminescence originates from a nonlinear anti‐Stokes process, where near‐infrared (NIR) excitation is converted into shorter‐wavelength emission. This unique property enables background‐free detection by eliminating optical interference from biological specimens or assay substrates. This advantage benefits from the inherently low autofluorescence of biological samples under NIR illumination,^[13]^ which substantially improves the signal‐to‐noise ratio and overall detection sensitivity.[^14^, ^15^] The relatively lower absorbance of NIR light by biological molecules, compared with UV/visible light, also minimizes photodamage to them. In addition, UCNPs exhibit exceptional photostability with testing results sustained for hours,[^16^, ^17^] which allows assays to provide reliable and reproducible readouts without signal degradation during extended handling or observation.[^18^, ^19^, ^20^] Despite their limited quantum yield, typically below 1%,[^21^, ^22^, ^23^] UCNPs remain sufficiently bright for quantitative assays, particularly when highly doped to achieve enhanced emission under powers above 10^2^ W/cm^2^.[^7^, ^12^] UCNPs have been widely applied for LFIAs, including detecting prostate, biomarker,[^12^, ^24^] cardiac troponin I,^[25]^ hepatitis B virus[^26^, ^27^] etc. We recently reported the use of UCNP‐based LFIAs for SARS‐CoV‐2 RAT, achieving a LOD of 10 pg/mL.^[7]^ We also demonstrated the strong potential of UCNP‐LFIAs for POCT when combined with a portable detection reader.^[6]^ Despite these advances, we recognized that the use of nitrocellulose membranes poses an inherent limitation in further lowering the LOD and improving detection accuracy.

To minimize non‐specific binding while maintaining a rapid turnaround time and other features of LFIAs, we here report a pipette tip‐based immunoassay approach (Beads‐on‐a‐Tip, as shown in Scheme 1b), where the magnetic beads (MBs) become the antigen capture substrate with UCNPs as the antigen reporting nanoparticles. MBs have the large surface area for antigen binding and enrichment, good dispersion in suspension for efficient reaction kinetics, and can be easily trapped in the pipette tip by magnets.^[28]^ The architecture of trapping MBs in the pipette tip not only enables the repeated washing of reagents but also facilitates the targeted capture, incubation and enrichment of target analytes at specific points of interest using a magnet, thereby minimizing non‐specific binding effects and improving the detection sensitivity as well as the analytical linear range, as illustrated in Scheme 1c.

In this work, as illustrated in Scheme 1b and Figure S1a, our Beads‐on‐a‐Tip design replaces nitrocellulose membranes (NCs) on the backing card, and the entire assay can be conducted within a 10 μL pipette tip that can be embedded into a custom‐designed cartridge adapter, so that it shares the existing upconversion strip reading device (Figure S2). By a magnet beneath the tip (indicated by the red circle in Scheme 1b), the immunocomplex of MBs are entrapped within a narrow region (As shown in Figure S1b), which takes the advantage of our recently reported geometric LFIA strip.^[7]^ This narrow region, smaller than 1 mm, matches the 400 μm beam size of the focused 980 nm excitation laser in our upconversion strip reader. The entire reaction was performed within disposable microtubes, which provides a homogenous reaction environment for reagent mixing. As shown in Scheme 1c, MBs are conjugated with capture antibodies (MBs‐Abs) to capture target antigens, and UCNPs are conjugated with detection antibodies (UCNPs‐Abs), forming a typical sandwich‐type immunocomplex (MBs‐Abs‐antigen‐Abs‐UCNPs). When an external magnet is applied, the unbound nanoparticles and non‐specifically bound components are removed, with a suspension condensed to 10 μL. After transferring the final immunocomplex into the pipette tip, magnetic enrichment takes place at the test zone, where the focused laser covers the MBs‐bound immunocomplex. The enrichment significantly enhances sensitivity, with a LOD towards sub‐pico‐gram range.

## 2 RESULTS

### 2.1 Successful preparation of UCNPs‐Abs and MBs‐Abs

**FIGURE 1:**
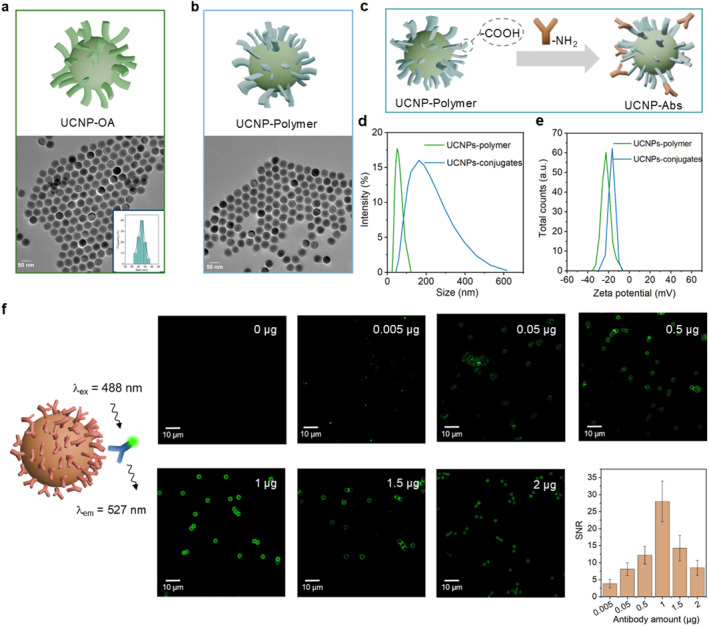
Step‐by‐step validation of successfully formed UCNPs‐Abs and optimization of antibody‐to‐bead ratio to form MBs‐Abs. (a) TEM image of the as‐synthesized UCNPs with oleic acid surface; (b) TEM image of the UCNPs after surface modification by using polymers; (c) Polymer coating introduces ‐COOH groups on UCNPs, enabling covalent conjugation with antibodies to form UCNPs‐Abs; (d, e) DLS and zeta potential measurements for UCNPs‐polymer and UCNPs‐Abs conjugates; (f) Alexa488‐labeled secondary antibodies were used to evaluate the amount of primary antibodies bound to the bead surface. Representative confocal images show varying fluorescence intensities per bead corresponding to different antibody input amounts (per 12 μg of beads). Statistical analysis was performed by quantifying the fluorescence intensity of at least 50 beads per group. DLS, dynamic light scattering; MBs‐Abs, magnetic beads are conjugated with capture antibodies; TEM, transmission electron microscopy; UCNPs‐Abs, upconversion nanoparticles are conjugated with detection antibodies.

UCNPs following the design of β‐NaYF_4_:40% Yb^3+^,4% Er^3+^@NaYF_4_ Core@shell structure were synthesized using the co‐precipitation method as reported elsewhere^[29]^ (see Supporting Information S1 for details). The upconversion emission spectrum shown in Figure S3 exhibits the characteristic Er^3+^ emission peaks in the green and red bandwidths. The transmission electron microscopy (TEM) result, shown in Figure 1a, exhibits a uniform spherical morphology with an average diameter of 43 nm. The monodispersity of UCNPs in cyclohexane is attributed to the surface‐capping ligand, oleic acid (illustrated in green in Figure 1a). To render the UCNPs compatible with the aqueous phase, we synthesized a reversible addition‐fragmentation chain transfer polymer (illustrated in blue in Figure 1b) to replace the oleic acid, following the procedure established in our previous work^[30]^ (see Supporting Information S1). After ligand exchange, the resulting UCNPs‐polymer complex remained monodispersed in water. Although the polymer layer is invisible under TEM due to the low electron contrast of its constituent elements, its presence was confirmed by fourier transform infrared (Figure S4), dynamic light scattering (DLS) (Figure 1d), and zeta potential (Figure 1e) measurements. The surface‐exposed ‐COOH groups of the polymer enable covalent coupling with ‐NH_2_ groups of the antibodies via (N‐ethyl‐N'‐(3‐(dimethylamino)propyl)carbodiimide (EDC) chemistry, resulting in UCNPs‐Abs conjugates, as illustrated in Figure 1c. After this bioconjugation, the zeta potential of the UCNPs shifted from −22.4 to −16.8 mV and the hydrodynamic size increased from 58 to 149 nm. While a slight broadening and an increase in average particle size were observed following antibody conjugation, this on one hand confirms the successful attachment of antibodies, and on the other hand suggests that some conjugates may have formed clusters.^[31]^ This phenomenon can be attributed to structural rearrangements of the antibodies upon adsorption, whereby partially unfolded domains expose hydrophobic or charged residues that mediate interparticle interactions, ultimately driving cluster formation.[^7^, ^32^]

In parallel, MBs‐Abs conjugates were prepared using the EDC coupling method (see Supporting Information S1). To determine the optimal antibody concentration that maximizes the average number of antibodies per bead with the aim of achieving the highest number of available reactive epitopes for the subsequent sandwich immunoreaction, a concentration gradient ranging from 0.005 to 2.5 μg per 12 μg of beads was evaluated (Figure 1f). The assessment was performed by adding a secondary antibody labeled with Alexa488, where the fluorescence intensity of Alexa per bead reflects the trend in the amount of active antibodies on the bead. Figure 1f shows a representative series of confocal images under 488 nm excitation. Statistical analysis of at least 50 beads per group revealed a saturation effect in the signal‐to‐noise ratio (SNR), defined as the emission intensity of Alexa488 per bead divided by the background noise. While between 0.005 and 1 μg of antibody input, the binding efficiency increased with the antibody concentration, a notable decrease in fluorescence intensity was observed when the antibody input exceeded 1 μg, likely due to the concentration quenching of Alexa dyes on the bead surface, rather than the possible antibody saturation,^[33]^ otherwise the fluorescence signal at 1.5 and 2 μg should have reached a plateau. In the following sections, when performing the Beads‐on‐a‐Tip assay using 1 and 1.5 μg antibodies (Figure S5), a lower LOD of 0.101 pg/mL was achieved by using 1 μg antibody, compared to a LOD of 0.883 pg/mL using 1.5 μg antibody.

### 2.2 Optimizing incubation time

**FIGURE 2:**
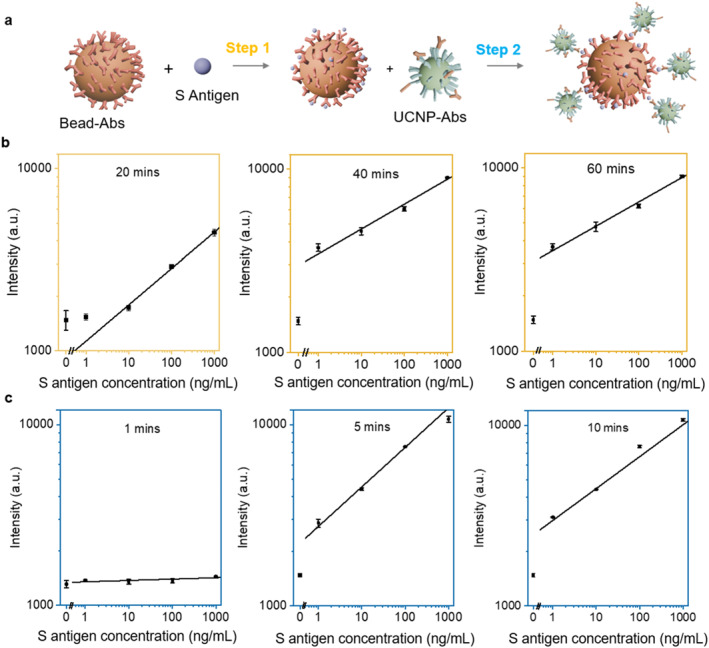
Optimization of incubation time for both MBs‐Abs antigen capturing and UCNPs‐Abs signal reporting steps. (a) The process of the two‐step formation of sandwich immunocomplexes; (b) Signal response curves showing the effect of different incubation times (20, 40, and 60 min) with S antigen (*n* = 3 in each time); (c) Signal response curves showing the effect of different incubation times (1, 5, and 10 min) with UCNP‐Abs (*n* = 3 in each time). MBs‐Abs, magnetic beads are conjugated with capture antibodies; UCNPs‐Abs, upconversion nanoparticles are conjugated with detection antibodies.

In the bead suspension assays, the lower number of MBs will benefit the effective analyte enrichment, as higher concentration of analytes will be captured by the lower number of MBs, but it will require longer time for the limited number of beads to capture the analyte molecules onto the surface of MBs. To find a reasonable balance between the enrichment efficiency and the analyte enrichment time, as illustrated in Figure 2a, we performed and optimized a two‐step reaction to form the sandwiched immunocomplex, including MBs‐Abs capturing the antigen and UCNPs‐Abs signal reporting. The incubation time was optimized to enhance antigen detection sensitivity, under a series of S antigen concentrations. Signal intensity increased consistently with antigen concentration across all groups, indicating effective concentration‐dependent capture and detection. At the MBs concentration of 12 μg/mL used in our following experiments, 20 min incubation was found insufficient, and extending the incubation to 40 min can significantly enhance the signal strength, which established a clear linear relationship from 1 to 1000 ng/mL. Negligible difference was observed for the incubation time between 40 and 60 min. Subsequently, at the UCNPs‐Abs concentration of 0.1 mg/mL, incubation durations for the UCNPs‐Abs signal reporting step at 1, 5 and 10 min were investigated by fixing the MBs‐Abs capturing incubation at the optimal 40‐min interval. As shown in Figure 2c, the signal intensity increased rapidly from 1 to 5 min across all the antigen concentrations, with a clear linear relationship. However, extending the incubation to 10 min did not yield any significant improvement, compared to the 5‐min condition.

### 2.3 Optimizing UCNPs‐Abs to beads‐Abs ratio

**FIGURE 3:**
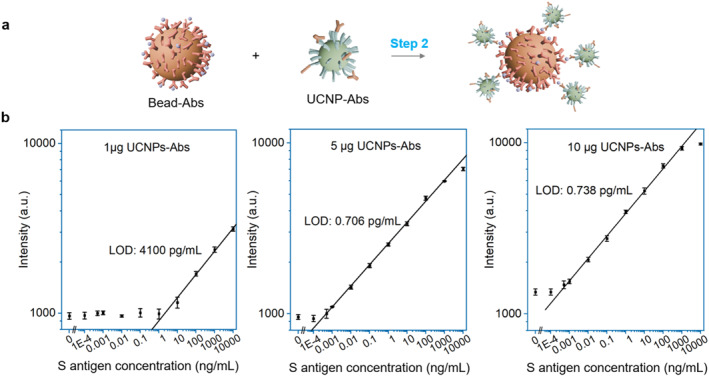
Effect of varied UCNP‐Ab concentrations on antigen detection. (a) Illustration of the second step in sandwich immunocomplex formation; (b) Calibration curves for S antigen detection using 1, 5, and 10 μg of UCNPs‐Abs (*n* = 10 in each variant). UCNPs‐Abs, upconversion nanoparticles are conjugated with detection antibodies.

UCNPs‐Abs serve as signal reporters whose signal strength should be proportional to the levels of antigen analytes, and their ratio to beads‐Abs plays a key role in modulating specific versus non‐specific binding during the assay. This ratio ultimately influences the LOD, which determines the sensitivity of the assay for detecting low‐abundance antigens. To optimize the parameter, we varied the ratio of UCNPs‐Abs to Beads‐Abs in Step 2 by testing 1, 5, and 10 μg of UCNPs‐Abs against a fixed amount of 2.4 μg Beads‐Abs. The assay was then performed across a broad range of antigen concentrations, from 0.0001 to 10,000 ng/mL, with results shown in Figure 3.

With 1 μg of UCNPs‐Abs, the assay exhibited limited upconversion response at lower antigen concentrations, with appreciable signal only at the concentrations higher than 10 ng/mL. This indicates that the limited amount of UCNPs‐Abs conjugates was insufficient for effective target recognition and signal readout, resulting in a relatively high LOD of 4100 pg/mL. LOD in this work is defined as the target concentration where the intensity is equal to the sum of the background noise and three times the standard deviation above the background noise.[^34^, ^35^] Increasing the number of UCNPs‐Abs conjugates to 5 and 10 μg has significantly extended the detectable range, reaching to the femtogram level. The enhanced upconversion signal at each antigen concentration suggests that a greater number of UCNPs‐Ab conjugates successfully bound to target antigens, thereby improving the assay's ability to detect trace analyte levels. This led to improved LODs of 0.706 and 0.738 pg/mL for the 5 and 10 μg conditions, respectively. The slightly higher LOD observed with the 10 μg condition is attributed to increased non‐specific binding, as evidenced by elevated signal intensities in the control samples. Therefore, 5 μg of UCNPs‐Aba conjugates was chosen in our protocol. Then, the repeatability of the assay under the final optimized conditions was evaluated through three independently performed experiments using the same batch of UCNPs. The DLS and zeta‐potential measurements of both polymer‐modified and antibody‐conjugated UCNPs showed minimal variation among the three replicates (Figure S6). Despite slight variations in signal intensity, the three independent replicates demonstrated good reproducibility of the assay (Figure S7). In the future real sample measurements, a blank control can be first tested to define the baseline signal. Given the established linear calibration relationship under the optimized conditions, the concentration of the unknown sample can then be approximately estimated accordingly.

### 2.4 Performance comparison with alternative detection strategies

**FIGURE 4:**
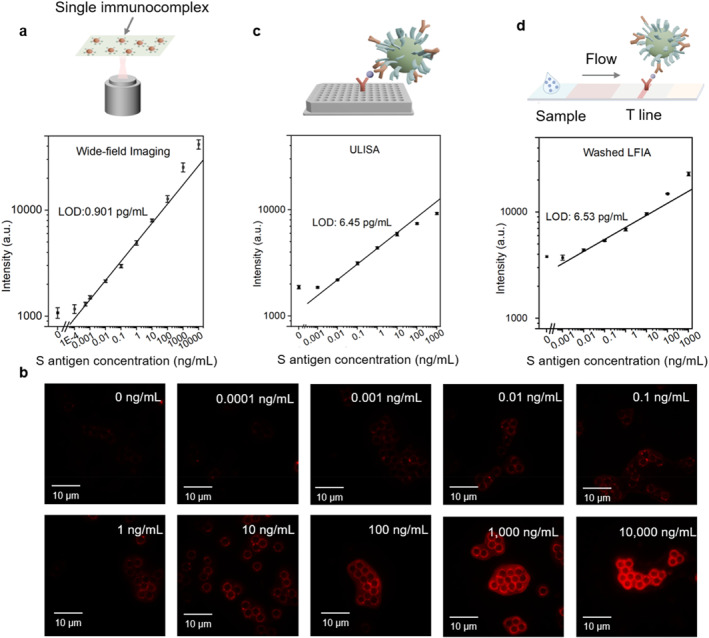
Comparative analysis of assay performance between three analytical methods and fluorescence images of sandwich immunocomplexes at varying antigen concentrations. (a) The calibration curves for S antigen detection using wide‐field imaging of beads (*n* =10); (b) Representative wide‐field fluorescence images of single immunocomplexes with varying S antigen concentrations, showing increased signal intensity with rising antigen levels; (c) The calibration curves for S antigen detection using ULISA (*n* = 10) and (d) washed LFIA (*n* = 10). LFIA, lateral flow immunoassay; ULISA, upconversion enzyme‐linked immunosorbent assay.

To illustrate the power of bead assays in terms of LOD and dynamic range, we performed the wide‐field imaging by using a purpose‐built upconversion microscope with 980 nm laser excitation to visualize individual beads and the homogeneity of their formed sandwich immunocomplexes. As presented in Figure 4b, the visualized results of the representative images across a wide concentration range (0–10,000 ng/mL) can quantitatively validate those results obtained from the Beads‐on‐a‐Tip assay, that is, using a 5 μg UCNPs‐Abs, a calculated LOD of 0.901 pg/mL was achieved, compared with LOD of 0.967 pg/mL and a LOD of 241 pg/mL achieved using a 10 and 1 μg UCNPs‐Abs, respectively (see Figure S8). The imaging results also confirm that individual beads exhibit monodispersed upconversion signals. Altogether, it can be concluded that our Beads‐on‐a‐Tip method is as sensitive as the imaging‐based approach, but more suitable for POCT by its simplicity in signal readout.

To assess the benefits of beads‐Abs for signal enrichment and active washing, we performed the assay using a microplate‐based upconversion enzyme‐linked immunosorbent assay (ULISA, Figure S10), where the formed Abs‐Ag‐UCNPs‐Abs sandwich immunocomplexes are randomly immobilized on the bottom of the well plate, and washing is performed passively by removing unbound species. ULISA provides a homogeneous liquid‐phase environment with incubation conditions similar to those in the POCT‐on‐a‐Tip platform. The results, shown in Figure 4c, demonstrate a LOD of 6.45 pg/mL via ULISA, which is approximately nine times higher than that achieved by the Beads‐on‐a‐Tip method.

As the Beads‐on‐a‐Tip platform is designed for POCT, it is important to evaluate its performance against the conventional paper‐based LFIA. To ensure sufficient binding, an off‐line reaction between the UCNPs‐Abs conjugates and target antigens was firstly carried out, followed by application of the reaction products onto the paper strip for lateral flow testing. Signal detection was performed at the test (T) line, and an LOD of 9.72 pg/mL was achieved (Figure S9). After a single wash with pure buffer, the best achievable LOD reached 6.53 pg/mL, as shown in Figure 4d.

## 3 DISCUSSION AND CONCLUSION

### 3.1 Analysis of achieved performance relative to literature benchmarks

Table 1 summaries the analytical performance of LFIA, microwell plate‐based, imaging, and Beads‐on‐a‐Tip methods achieved in this study, and compares the representative works reported by other groups in (SARS‐CoV‐2) antigen detection, in terms of the detection range, LOD and point‐of‐care applicability.

**TABLE 1:** Comparison of the analytical performance of various immunoassay platforms, including LFIA‐based methods, microwell plate assays, wide‐field imaging, and our Beads‐on‐a‐Tip system.

Methods	Detection range	LOD (pg/mL)	Point‐of‐care	Refs
LFIA	10 pg/mL−1 μg/mL	6.53	Yes	This work
	1 ng/mL−1 μg/mL	38		[36]
	50 pg/mL−1 μg/mL	33		[37]
	100 pg/mL–10 μg/mL	10		[7]
Microwell plate	10 pg/mL−1 μg/mL	6.45	No	This work
	Up to 10 ng/mL	30		[38]
	1 pg/mL−100 ng/mL	1.3		[39]
Imaging	500 fg/mL−10 μg/mL	0.901	No	This work
	100 fg/mL −100 ng/mL	2.7		[39]
	200 fg/mL−20 μg/mL	0.00073		[40]
**Beads‐on‐a‐Tip**	**500 fg/mL–10 μg/mL**	**0.706**	**Yes**	**This work**

Abbreviation: LFIA, lateral flow immunoassay.

In LFIA, Oh et al. reported a strategy involving the formation of plasmon color‐preserved AuNP clusters by assembling streptavidin‐coated AuNP cores with satellite AuNPs functionalized with biotinylated antibodies.^[36]^ This configuration achieved a LOD of 38 pg/mL in detecting N antigen. In another study, Han et al. created a SiO_2_@Au/quantum dots hybrid structure, in which quantum dots and AuNPs were co‐assembled onto a silica core to enable dual‐mode colorimetric and fluorescent signal readout, resulting in a LOD of 33 pg/mL in detecting S antigen.^[37]^ By using UCNPs as the reporter, we have recently advanced the design of a geometric paper strip that minimizes the immunoreaction area for antigen detection achieving a LOD of 10 pg/mL^[7]^ and a LOD of 6.53 pg/mL in detecting N and S antigen analytes, respectively.

Microwell plate‐based setup offers longer incubation times for sandwich immunoreactions and suitable for washing steps. Huang et al. reported a one‐step assay using nanoplasmonic sensors integrated into standard 96‐well microplates, achieving a LOD of approximately 370 virus particles/mL, corresponding to about 30 pg/mL of N antigen.^[38]^ Gorris et al. developed a ULISA system that combined UCNPs with biotinylated antibody pairs in a conventional sandwich immunoassay format, achieving a LOD of 1.3 pg/mL and a broad dynamic range from 1 pg/mL to 100 ng/mL in detecting N antigen.^[39]^ Similarly, our ULISA setup has achieved a comparable LOD of about 6.45 pg/mL in this work.

Fluorescence imaging can achieve exceptional sensitivity towards a single‐molecule sensitivity. Gorris et al. examined a microscopic imaging approach based on the same immunocomplexes formed in the above mentioned microplate wells, achieving a LOD of 2.7 pg/mL.^[39]^ Compared to the upconversion microplate reader used in their ULISA format, the imaging‐based readout yielded approximately two‐fold higher signal intensity, along with an extended detection range that reached down to a level of 100 fg/mL. Li et al. reported a novel fluorescence‐linked immunosorbent assay based on streptavidin‐modified transparent 96‐well microplates and carboxylated fluorescent microsphere‐conjugated monoclonal antibodies by wide‐field fluorescence microscopy, resulting in an exceptionally low LOD of 0.00073 pg/mL for SAS‐Cov‐2 N antigen detection and a broad detection range of 0.02 pg/mL–20 μg/mL, with the saturation point at 200 ng/mL.^[40]^ According to the report, the high sensitivity was attributed to the use of biotinylated antibodies and the surface treatment of the microplates by the plasma and subsequent streptavidin modification that increases the binding efficiency of biotinylated capture antibodies while maintaining immunoreactivity.

In our work here, MBs can enrich analytes and the immunofluorescence assays conducted in the suspension facilitate the long‐term stability of conjugates. By replacing the nitrocellulose membrane with beads in a tip, the active washing overcomes the limitation of the passive capillary fluidics in nitrocellulose membranes that are often associated with non‐specific bindings and high background noise, as well as reporting nanoparticles' aggregation and their loss in the binding efficiency. Despite the LOD of 0.901 pg/mL achieved by the following fluorescence imaging, the system requires a bulky microscope, hard for POCT applications. The Beads‐on‐a‐Tip system developed in this work achieved a broad dynamic range (500 fg/mL to 10 μg/mL) and a low LOD of 0.706 pg/mL, representing a substantial sensitivity improvement over conventional LFIAs and microwell‐based methods. Notably, this platform technology takes advantage of the high sensitivity and low non‐specific binging of imaging approaches and the applicability of LFIAs towards more accurate and quantitative POCT. Future work will improve the conjugate stability through optimized surface chemistry and storage strategies, as well as incorporating automated fluid handling with active washing to enhance robustness and reproducibility in the assay. Moreover, the successful clinical performance previously achieved with our UCNP‐LFIA in real saliva samples further supports the strong clinical potential of the Beads‐on‐a‐Tip system, given its markedly broader detection range and substantially lower LOD.

## 4 EXPERIMENTAL SECTION

### 4.1 Reagents and buffers

YCl_3_·6H_2_O (99.99%), YbCl_3_·6H_2_O (99.99%), ErCl_3_·6H_2_O (99.99%), NaOH (98%), NH_4_F (99.99%), 1‐octacene (ODE), oleic acid (OA), tetrahydrofuran (THF), 2‐(N‐morpholino)ethanesulfonic acid (MES) powder, polyvinylpyrrolidone (PVP), sodium chloride solution, Trizma base (≥99.9%), Tween‐20, bovine serum albumin (≥96%), sucrose (≥99.5%) were purchased from Sigma‐Aldrich Pty Ltd. Dynabeads™ M‐270 carboxylic acid (14305D) was purchased from Thermofisher. AnteoTech Nano Kit (A‐PCKS) was purchased from AnteoTech. Sample pad (Whatman CF4, 22 mm × 50 m) and nitrocellulose membrane (20 mm × 50 m) were purchased from Cytiva Pty Ltd. The conjugation pad (254 mm × 0.3 m) was purchased from Biotech Australia Pty Ltd. Absorption pad (15 mm × 100 m) was purchased from Alcolizer Pty Ltd.

### 4.2 Antibodies and antigens

SARS‐CoV‐2 spike monoclonal antibody (40589‐D008), SARS‐CoV‐2 omicron spike receptor‐binding domain (RBD) specific antibody (40589‐D007) and SARS‐CoV‐2 omicron (XBB.1.16) spike S1 + S2 trimer protein (40589‐V08H48) were purchased from Sino Biological.

### 4.3 Synthesis of NaYF_4_:40%Yb^3+^, 4%Er^3+^ core nanoparticles

The NaYF_4_:40%Yb, 4%Er core nanoparticles were synthesized based on the co‐precipitation method. The liquid form is essential for controlling nucleation, growth, and monodispersity while preventing aggregation. In a typical procedure, 1 mmoL LnCl_3_·6H_2_O (Ln = Y, Yb, Er) with a molar ratio of 56:40:4 was added to a 50 mL three‐neck round‐bottom flask containing 6 mL OA and 15 mL ODE. The mixture was heated to 170°C under inert gas with a magnetic stir rotating at 500 round per minute. After maintaining for 40 min, the solution was cooled to room temperature (RT) to obtain a clear and transparent solution. Then, 10.5 mL methanol solution of NaOH (2.5 mmoL) and NH_4_F (4 mmoL) were added to the solution and stirred for 30 min at RT. The temperature was then slowly heated to 80°C and maintained for 30 min to evaporate excessive methanol until the bubbles disappeared in the solution. Next, the mixture was heated to 110°C and maintained for 10 min to remove residual methanol and water. In the final step, the solution was further heated to 300°C and maintained for 90 min. The resulting core nanoparticles were collected after cooling to RT and re‐dispersed in 5 mL cyclohexane after washing with cyclohexane/ethanol several times.

### 4.4 Synthesis methods of shell precursor for core‐shell spherical nanoparticles

The shell precursor was prepared for the epitaxial growth of the shell onto the core nanoparticles through the hot‐injection method. The synthesis of shell precursor was similar to that of the core as described above. For the shell precursor, the final step was to cool down the solution to RT after removing residual methanol and water. The spherical nanoparticles in this chapter were coated with an inert shell; therefore, the starting material of Ln^3+^ ion was 1 mmoL of YCl_3_·6H_2_O.

### 4.5 Synthesis of NaYF_4_:40%Yb^3+^, 4%Er^3+^@NaYF_4_ core@shell nanoparticles

The core@NaYF_4_ core‐inert shell spherical nanoparticles were synthesized using the epitaxial shell growth method. Firstly, 0.2 mmoL of the as‐synthesized core nanoparticles dissolved in cyclohexane was added to the flask with 6 mL of OA and 6 mL of ODE. The solution was sealed and degassed by inert gases and then heated to 80°C to remove cyclohexane with a magnetic stir rotating at 800 round per minute. After the complete removal of bubbles, the solution was further heated to 300°C. The shell precursor in the 3‐mL syringe with a minimum scale of 0.1 mL was injected into the solution at the rate of 0.2 mL/3 min, during which the temperature was always maintained at 300°C. The total amount of shell precursor was based on the size of the core and the desired size of the final core‐shell. Once finishing the hot‐injection cycles, the solution was further kept at 300°C for 10 min for complete nucleation and then cooled down to RT. The following washing protocol was the same as that of the core nanoparticles.

### 4.6 UCNP‐polymer modification

The UCNP concentration was quantified by weighing the dried nanoparticles after evaporating a known volume of the dispersion. The ligand exchange method used copolymer (5 mg) dissolved in THF (0.5 mL) first and then sonicated. Then, UCNPs (5 mg) were added to the dispersed solution, making a total volume 2 mL. The mixture was sonicated until mixed and incubated in a shaker at 600 rpm at RT for 12 h. The polymer‐coated UCNPs were washed with THF, THF‐H_2_O (1:1 in volume), and H_2_O, respectively, by centrifugation at 20240 g for 30 min and re‐dispersed in H_2_O.

### 4.7 Conjugation of omicron antibody on the surface of UCNPs‐polymer (modified UCNP‐Ab)

AnteoTech Nano Kit (A‐PCKS) was used for bioconjugation of the UCNP‐polymer with detection antibodies (omicron spike RBD antibody). In brief, 12 μL UCNP‐polymer (10 mg/mL) was mixed with 100 μL activation buffer containing 25 mM sodium fluoride and incubated on a shaker (800 rpm) for 1 h at RT. Then, the active nanoparticles were washed with particle washing solution (200 μL) once and then conjugation solution (200 μL) by centrifugation at 20240 g rpm for 1 h at 4°C. Subsequently, 200 μL of conjugation buffer was added. Then, 100 μL active UCNP‐polymer solution containing 25 mM sodium fluoride was mixed with 4 μL of omicron spike RBD antibody (1 mg/mL). The mixture was incubated on a shaker (800 rpm) for 1 h at RT. Next, 10 μL of BSA solution (10% w/w) was added to the mixture, followed by another 1‐h incubation. The omicron spike antibody‐UCNPs (UCNPs‐Abs) were then centrifuged at 20240 g for 1 h at 4°C and washed with the storage buffer. Finally, the UCNPs‐Abs were resuspended in 100 μL of storage buffer and stored at 4°C for further use.

### 4.8 The fabrication of test strips and procedure of LFIA test

This lateral flow assay used only one T‐line design. The test line area of the nitrocellulose membrane was sprayed with the antibody (0.125 mg/mL) using a dispenser and dried for approximately 1 h at 35°C. Then, the nitrocellulose membrane, conjugation pad, sample pad, and absorption pad were mounted on the PVC back pad orderly with 2 mm overlap. The assembled pads were cut into strips with a width of 3.3 mm and assembled in the cartridges.

In the strip testing, 1 μL of UCNP‐Abs (1 mg/mL) was transferred to a 150 μL running buffer (10 mM Tris buffer, pH 7.6, with 1% BSA and 0.6% Tween‐20) with different concentrations of the antigen (0, 0.001, 0.01, 0.1, 1, 10, 100, 1000 ng/mL). For the unwashed LFIA, after mixing the samples for 5 min, 50 μL of the sample solution was added to the sample pad of the strip. For the washed LFIA, after dropping 50 μL of sample solution, the strips were washed with 100 μL of running buffer. After 5 min, the strip was scanned on the test line by our strip reader (Lasteck Photonic Technology Solutions) equipped with 980 nm laser. The emission intensity at 650 nm of the nanoprobes at the test line area of the NC membrane was then analyzed.

### 4.9 The procedure of ULISA assay

The microplate was coated with 5 μg of SARS‐CoV‐2 spike monoclonal antibody in 10 mM PBS and incubated overnight at 4°C. The next day, the plate was washed three times with PBS containing 0.05% Tween‐20 and then blocked with PBS with 2% BSA for 2 h at RT. After blocking, the plate was washed again, followed by the addition of serial dilutions of antigens, which were incubated for 1 h. The plate was then washed three times. Subsequently, UCNP‐conjugated antibodies (omicron variant spike RBD) were diluted to 40 μg/mL with the storage buffer (10 Mm Tris buffer, pH 7.4, 150 mM NaCl, 1% BSA, 0.5% sucrose, 25 mM NaF, 1% Tween, 0.9% PVP) then added and incubated for another 1 h. After a final washing step, the plate was dried completely. The results were measured using a customized 980 nm microplate reader.

### 4.10 Conjugation of MBs‐Abs

The EDC‐N‐hydroxysuccinimide (NHS) method was used for the bioconjugation of MBs with capture antibodies. First, the MBs were washed three times by adding 100 μL of 25 mM MES, pH 5, and mixing well. After washing, 0.3 mg of beads (30 mg/mL) was transferred to a reaction tube and concentrated to generate a 100 mg/mL solution. EDC and NHS were each separately dissolved in 25 mM MES buffer (pH 5) at a concentration of 40 mg/mL. Then, 25 μL of the EDC solution and 25 μL of the NHS solution were added to the washed MBs. The mixture was gently mixed and incubated with slow tilt rotation at RT for 40 min. The following activation, the tube was placed on a magnet for 1 min to separate the beads, and the supernatant was removed. The MBs were then washed three times with 200 μL of 25 mM MES buffer, pH 5. Then suspend the activated MBs to 25 μL of MES buffer. Then, take 5 μL of solution and 1 μg of capture antibodies dissolved in 25 mM MES buffer (pH 5) were added to the activated MBs, adjusting the final volume to 50 μL. The mixture was incubated for 1 h at RT with slow tilt rotation. After antibody conjugation, the tube was placed on a magnet for 1 min, and the supernatant was discarded. The MBs‐Abs were then washed three times with washing buffer (10 mM phosphate buffer, pH 7.4 containing 0.05% Tween‐20). Subsequently, 1% BSA was added for blocking, and incubated for 40 min. Finally, the MBs‐Abs were washed three times with washing buffer to complete the conjugation process and stored in 25 μL 10 mM phosphate buffer.

### 4.11 The procedure of MBs‐based assay

Initially, washed beads are functionalized with specific antibodies to form the conjugated MBs. Between the individual steps, the microtubes were placed on magnetic holders to separate and wash the formed MB‐bound immunocomplexes from unbound reagents and the sample complex. Upon incubation, the target antigens with different dilutions (0–10,000 ng/mL) bind to the antibodies on the bead surface, forming the conjugated MBs‐Ags complex. The conjugated UCNPs, are then introduced, binding to the antigen to form a sandwich‐format complex. The MBs‐UCNPs bioconjugates could be used to bind the analyte and separate it from the diluted samples by applying an external magnet.

### 4.12 Detector and software

The detector was a custom‐built system that comprised dichroic mirrors, lenses, and a spectrometer. Spectrum software for intensity recording was provided by Lastek Pty Ltd. Both LFIA and MBs‐based assays use the same detector and software. The ULISA assay uses a 980 nm microplate reader with the same components of detector and software.

### 4.13 Confocal microscope

An inverted confocal microscope (FV1200 Olympus) was combined with a self‐built wide‐field microscope connected to a butterfly FBG‐stabilized 976 nm laser diode (900 mW Thorlabs). The filter cube for the detection of Er^3+^‐doped UCNPs consisted of a dichroic mirror (980 nm customized) and a short‐pass filter (842 nm Thorlabs). The images were acquired on an EMCCD camera (iXon Ultra 888 Andor) with a 100× objective (1.45 NA Olympus) and a 40× objective (1.4 NA Olympus), which resulted in a power density of 3.2 × 10^4^ W/cm^2^.

## AUTHOR CONTRIBUTIONS

Ziwei Wu and Jiajia Zhou were responsible for the investigation, including conducting the experiments, designing and validating the methodology, analyzing and presenting the data, and contributing to the drafting of the manuscript. Ziwei Wu, Jiajia Zhou and Dayong Jin participated in the experimental design and discussions. Yangjian Cai was responsible for the nanoparticle synthesis and Yitong Zhao was responsible for the microscopy set up. Mahnaz Maddahfar participated in the nanoparticle modification. Mohammad Sadraeian participated in the platform design. Jiajia Zhou and Dayong Jin were responsible for funding acquisition, project supervision, and the review, editing, and finalization of the manuscript.

## CONFLICT OF INTEREST STATEMENT

The authors declare no conflicts of interest.

## ETHICS STATEMENT

The authors confirm that no human or animal subjects were involved in this study.

## CONSENT STATEMENT

This study did not involve human participants, and informed consent was therefore not required.

## Supporting information

Supporting Information S1

## Data Availability

The data that supports the findings of this study are available in Supporting Information S1 of this article.
