# Supplementary material for: Beads‐on‐a‐Tip testing for ultrasensitive antigen detection across a large dynamic range
Source: Smart Mol. 2026 Jan 5;4(1):e70036. doi: 10.1002/smo2.70036 (PMC13104115; doi:10.1002/smo2.70036)
Supplement: Supplementary file 1 — Supporting Information S1 [file SMO2-4-e70036-s001.docx]

**SI**


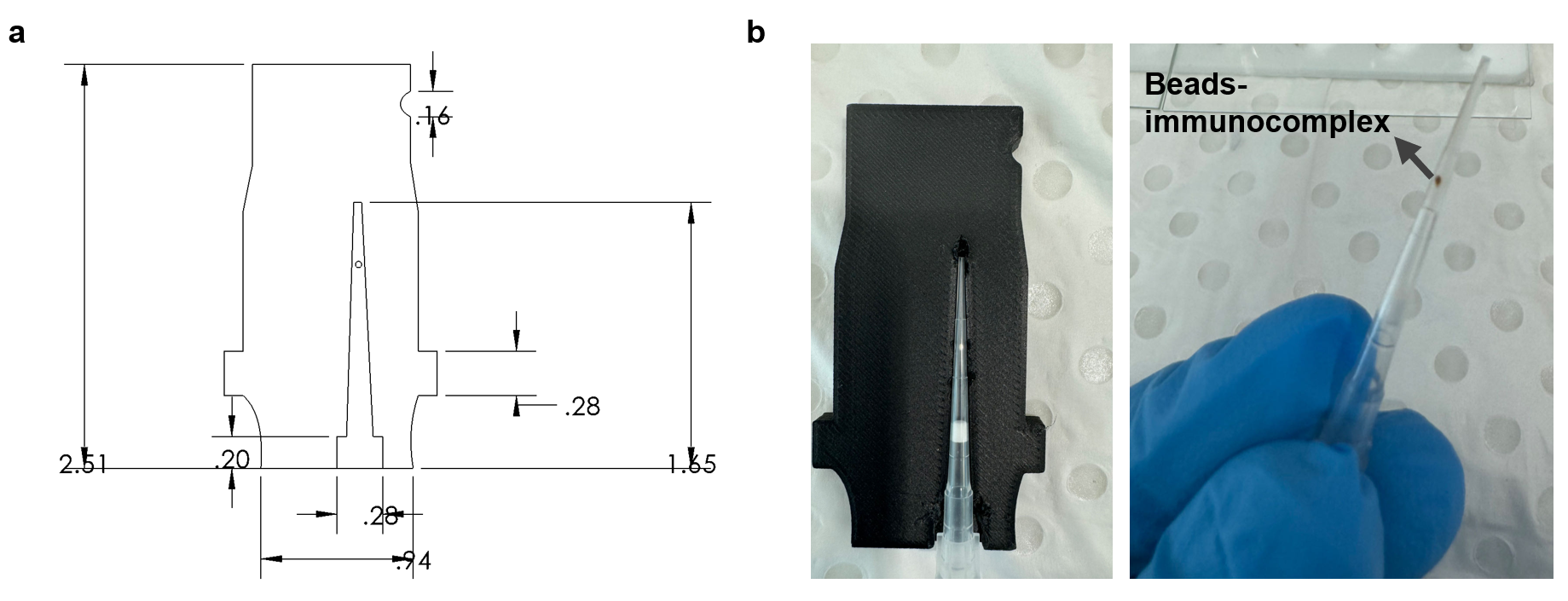


Figure S1. Scheme of Lab-on-a-Tip based platform design and implementation. a, CAD drawing of the Lab-on-a-Tip based holder (dimensions in cm); b, left: photo of the platform setup, right: photos of conjugated MBs and supernatant separation using magnet


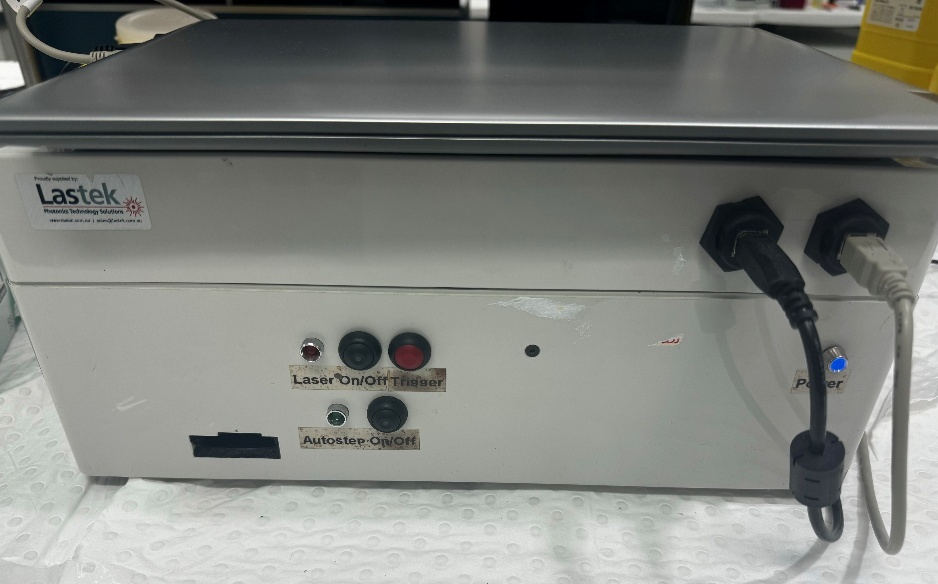


Figure S2. A portable detection reader was developed for the Lab-on-a-Tip platform, consisting of a laptop for spectral acquisition and analysis, and a optical detector integrated at the base of the platform.

Figure S3. The upconversion emission spectrum showing a green emission peak at 545 nm and a red emission peak at 650 nm.


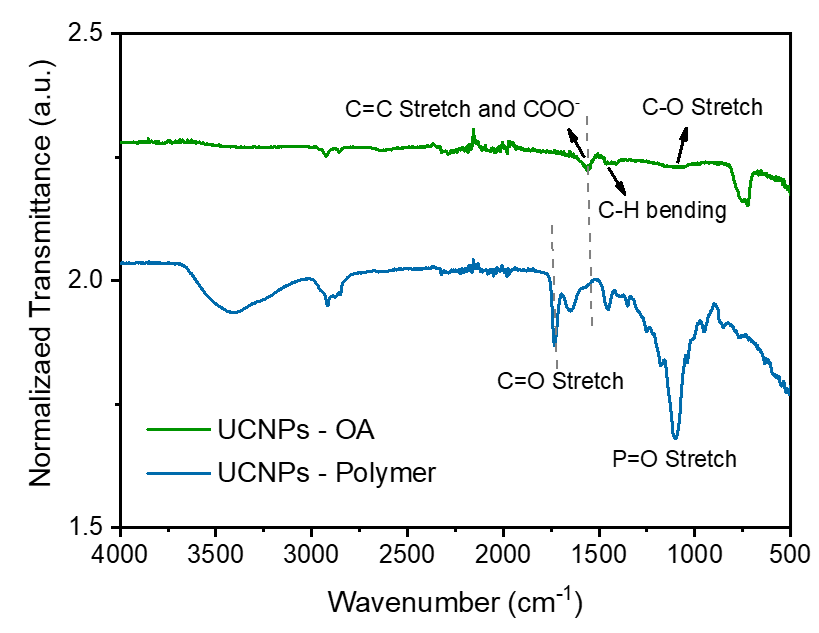


Figure S4. FTIR spectra of UCNPs-OA (green line), UCNPs-polymer (blue line). The disappearance of carboxylate signals at 1547 cm^−1^, and appearance of the C=O stretches at 1734 cm^−1^ and P=O stretches at 1105 cm^−1^, confirming the polymer grafting on the surface of UCNPs.


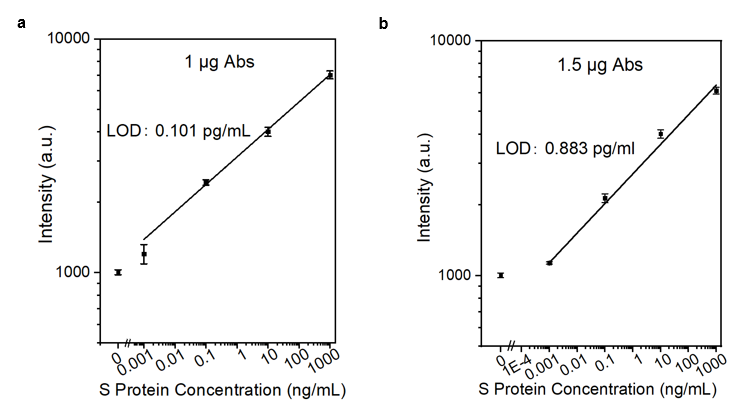


Figure S5. Calibration curves of assay performance with 1 μg and 1.5 μg antibody loading on MBs.


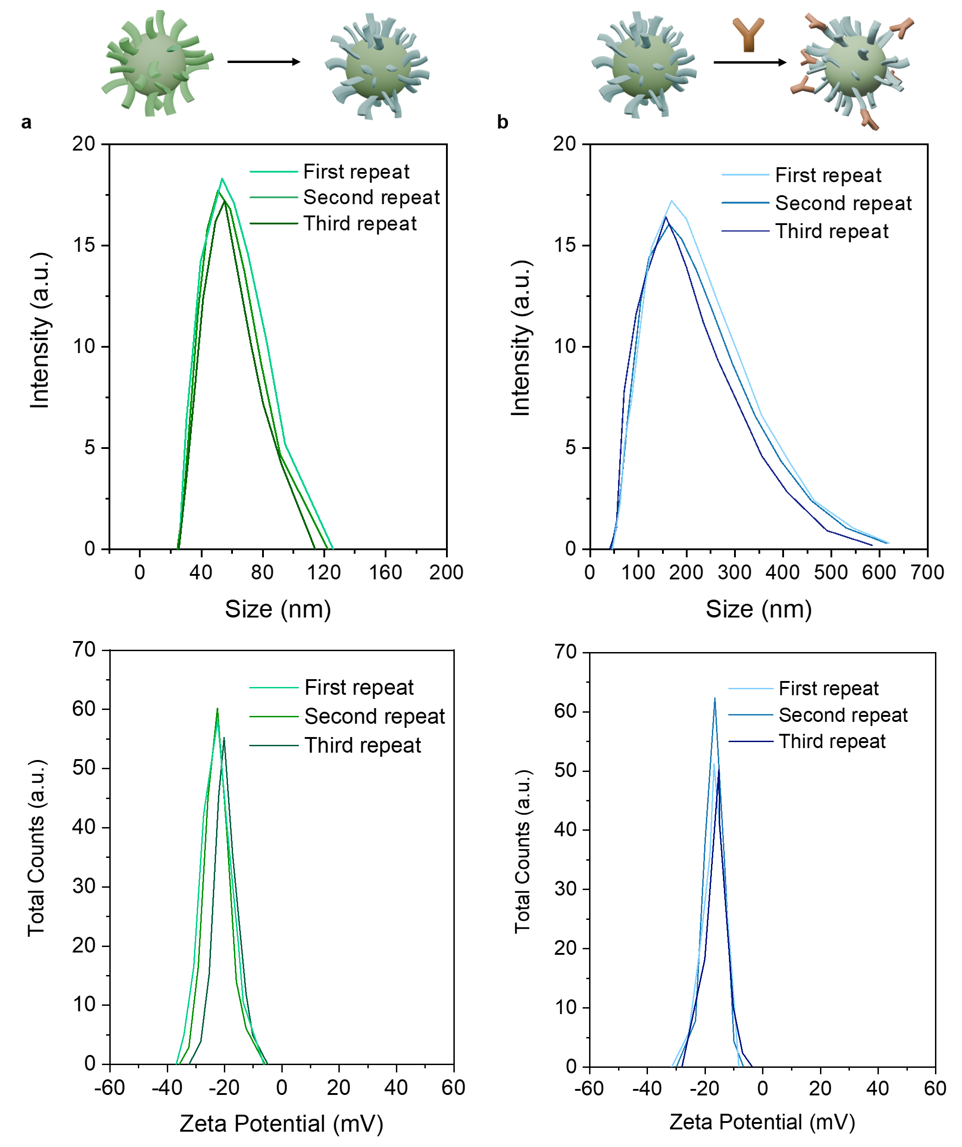


Figure S6. Repeatability of polymer modification and antibody bioconjugation on UCNPs (n=3). a, DLS and zeta-potential measurements of polymer-modified UCNPs; b, DLS and zeta-potential measurements of antibody-conjugated UCNPs.


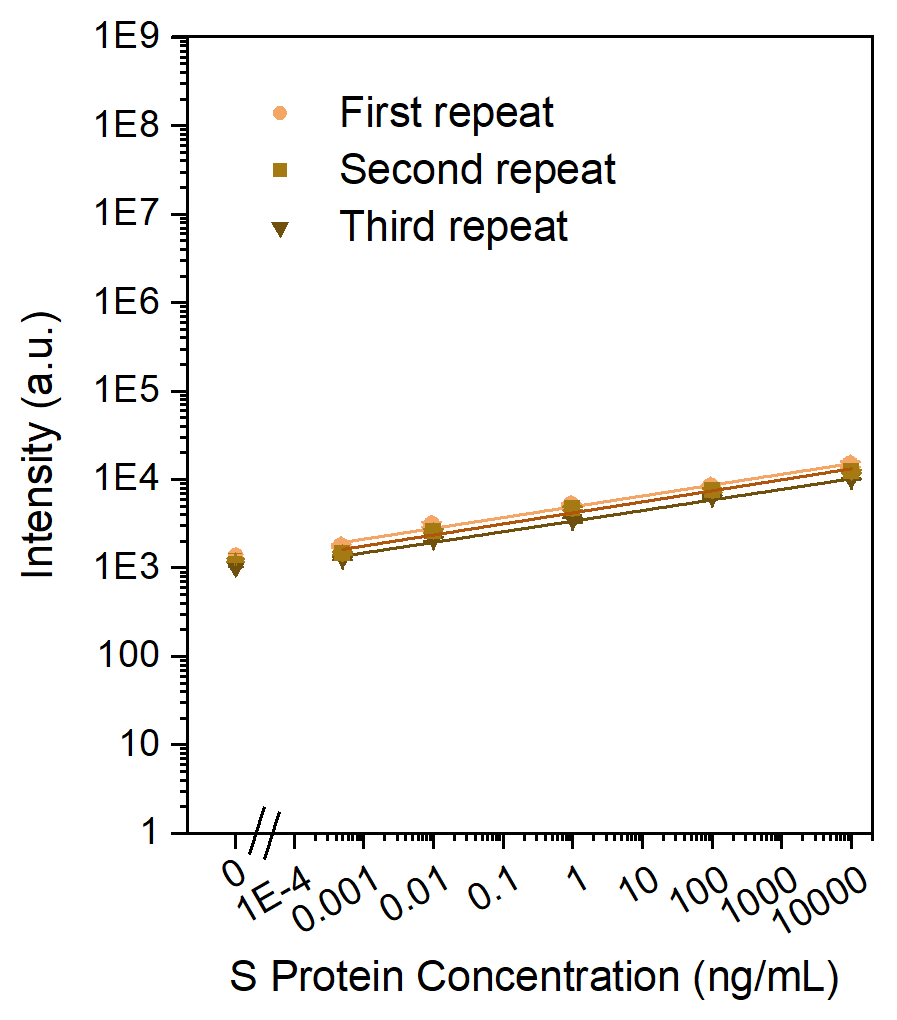


Figure S7. Calibration curves from three independent replicates of the beads-based assay (n=3).


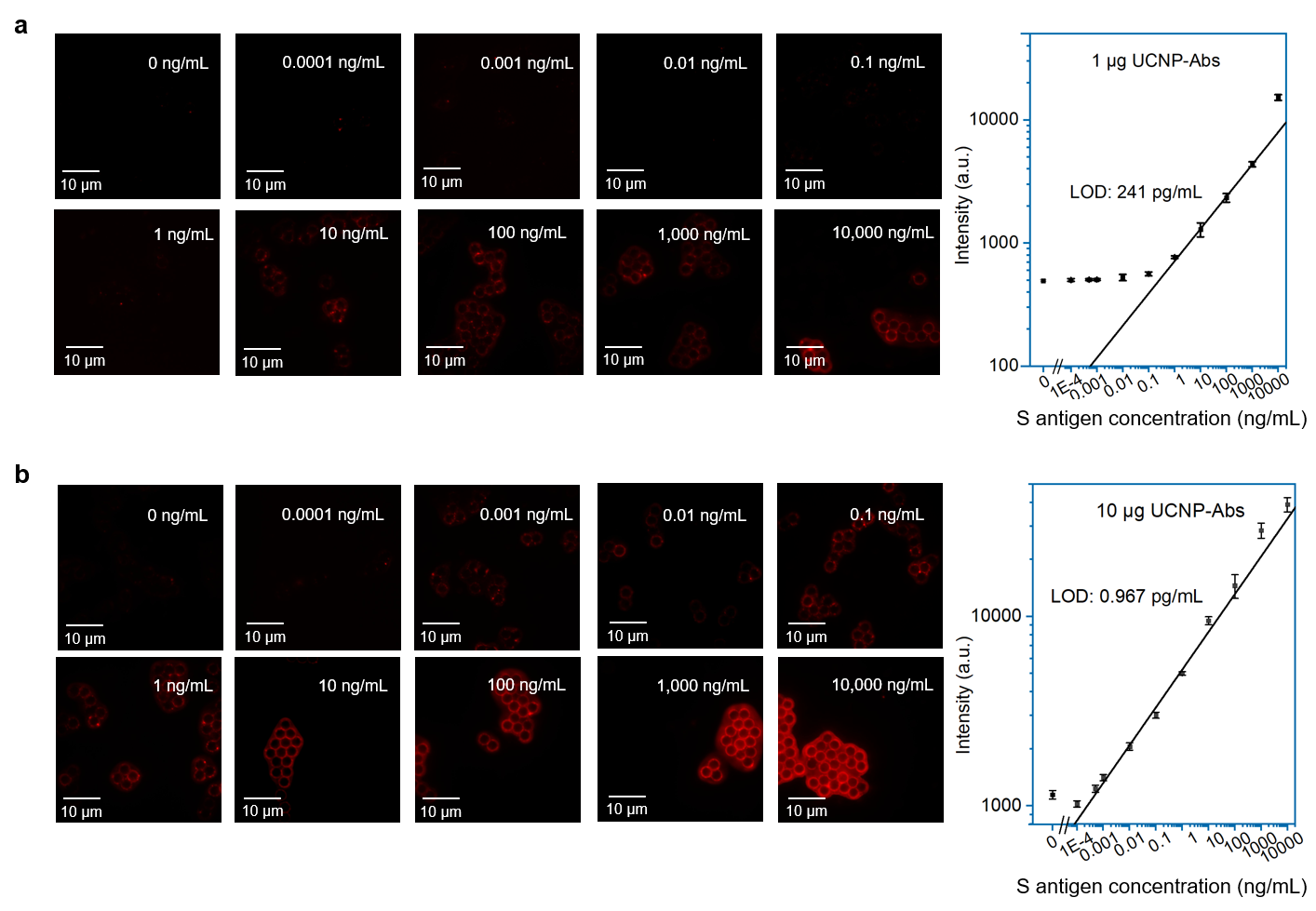


Figure S8. Fluorescence imaging under 980 nm wide-field imaging system and calibration curve of sandwich immunocomplexes at varying protein concentrations using 1 μg (a) 10 μg (b) of UCNPs-Abs conjugates.


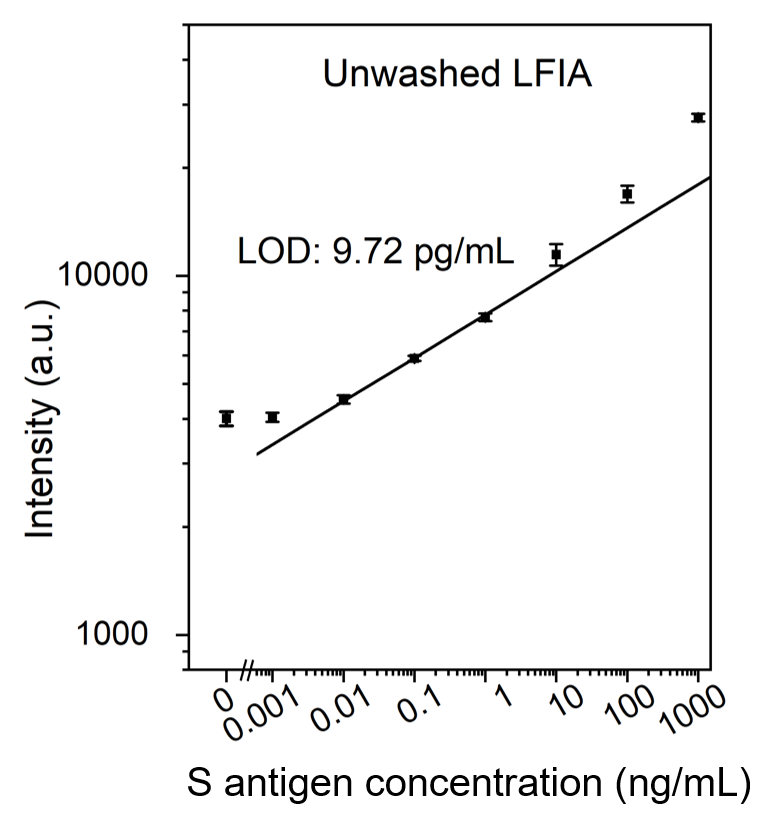


Figure S9. The result of the LFIA without washing


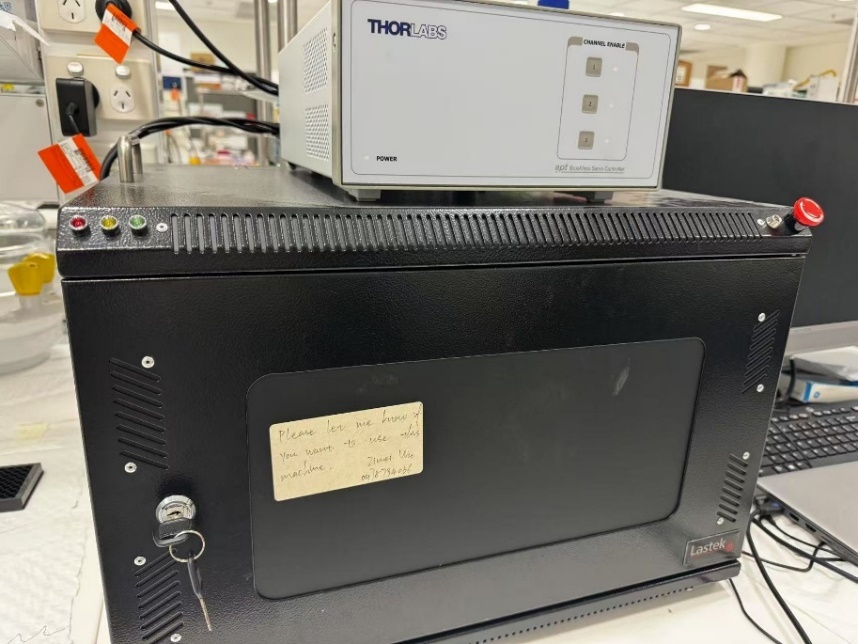


Figure S10. The customized 980 nm ULISA plate reader has a spectrometer on the top, and the detector for single UCNP-conjugates in the microplate is at the bottom.
